# SOCIAL INVISIBILITY OF ELDER ABUSE IN FAMILIES: A PHENOMENON IN PHILIPPINE SOCIETY

**DOI:** 10.1093/geroni/igae098.0954

**Published:** 2024-12-31

**Authors:** Carmelina Oyales, Victoria Apuan

**Affiliations:** Miriam College and Eunoia Educational Consulting, Quezon City, National Capital Region, Philippines; Miriam College, Quezon City, Rizal, Philippines

## Abstract

In a country where the prevailing culture is to give respect and care for the older persons, elder abuse is an interesting phenomenon. The study focuses on elder abuse and the different forms of abuse occurring in Filipino communities. The authors undertook a qualitative study composed of individual interviews of ten victims of elderly abuse. The aim was to explore the thoughts, feelings, sense of self, family dynamics and coping strategies of the abused. Further, data on the socio-cultural and economic risk and protective factors in the community and local government, the perspectives of thirty two members of local government and social welfare institutions were gathered through focus group discussions in five localities. Narrative and content analysis yielded the following

**results:**

that elder abuse is a multifaceted and complex experience for the victims. The research study shows that the victims suffered initial shock, indignation, depression and learned helplessness. The most frequent forms of abuse were emotional and psychological abuse, neglect, abandonment and financial exploitation. Poverty and ageism were an exacerbating factor. Actions to access local support mechanisms and interventions were absent or delayed due to the secrecy of the families regarding the phenomenon, as well as the lack of reporting and monitoring mechanisms on the community level. The invisibility of older adults abuse in families needs attention and plans for action to help elder abuse victims. Keywords: elder abuse, aging population, ageism, poverty, senior citizens human rights

